# Supplementary figures and images for: Drinking Warm Water Promotes Performance by Regulating Ruminal Microbial Composition and Serum Metabolites in Yak Calves
Source: Microorganisms. 2023 Aug 16;11(8):2092. doi: 10.3390/microorganisms11082092 (PMC10459242; doi:10.3390/microorganisms11082092)

**A****Permutation testing** $R^2=(0,0.9629)$ ,  $Q^2=(0,0.0141)$ 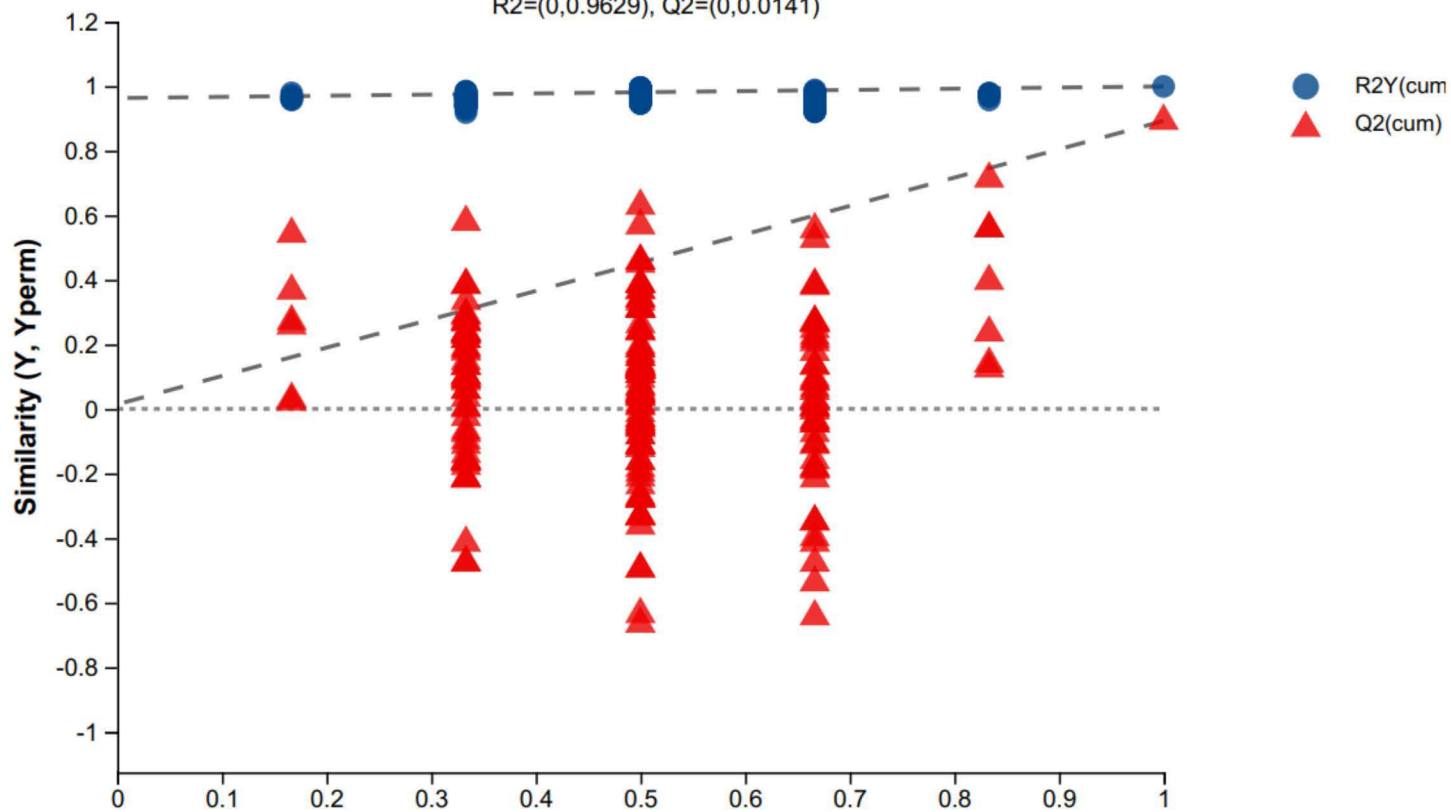**B****Permutation testing** $R^2=(0,0.9379)$ ,  $Q^2=(0,0.049)$ 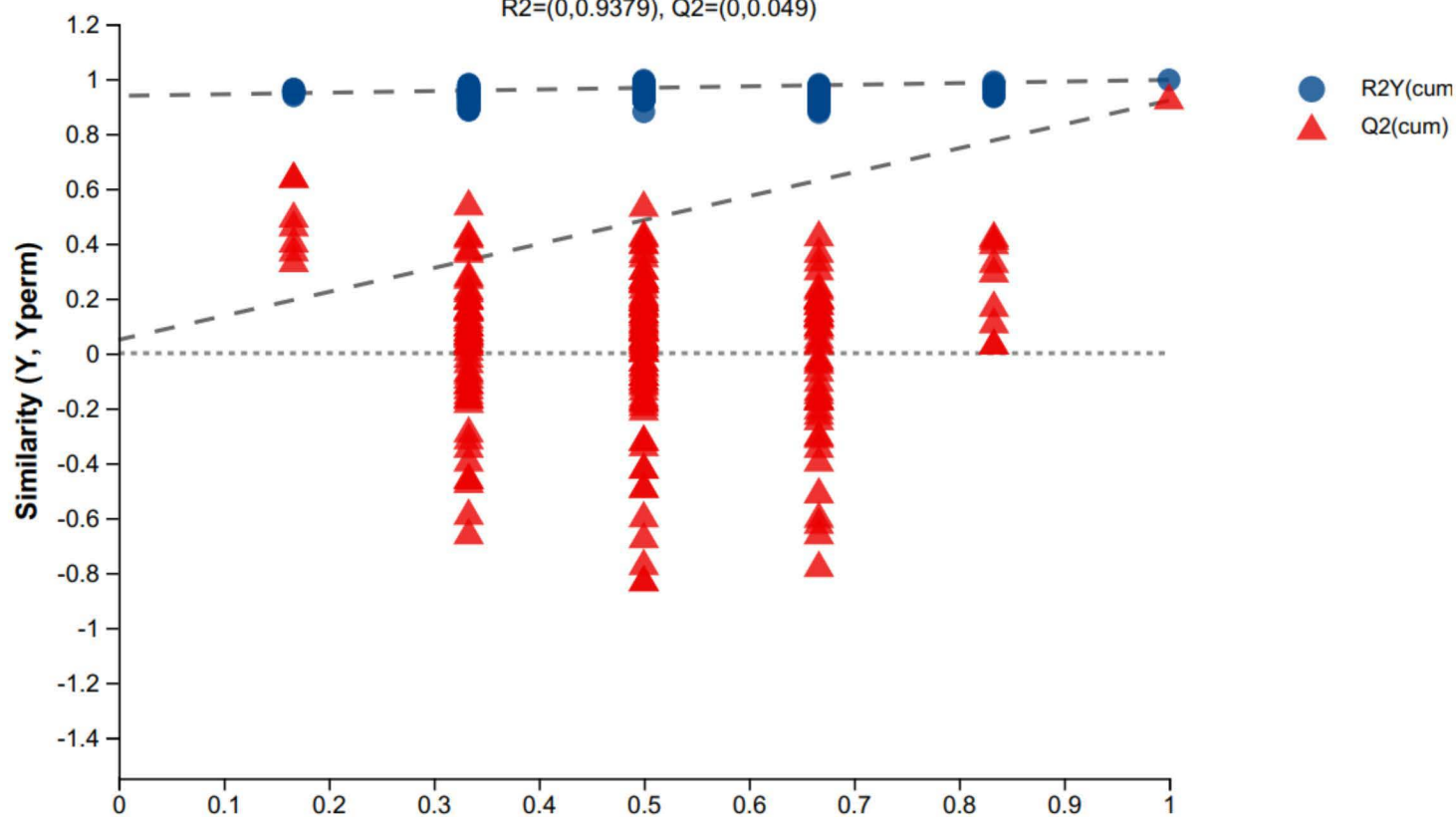

Supplement: Supplementary file 1 [file microorganisms-11-02092-s001.zip › microorganisms-2520507-supplementary/╨┬╜¿╬─╝■╝╨/Figure S1.pdf]
